# Supplementary material for: Differential Modulation of the Phosphoproteome by the MAP Kinases Isoforms p38α and p38β
Source: Int J Mol Sci. 2023 Aug 4;24(15):12442. doi: 10.3390/ijms241512442 (PMC10419006; doi:10.3390/ijms241512442)
Supplement: Supplementary file 1 [file ijms-24-12442-s001.zip › ijms-2492061-supplementary.pdf]

Supplementary Figure legends

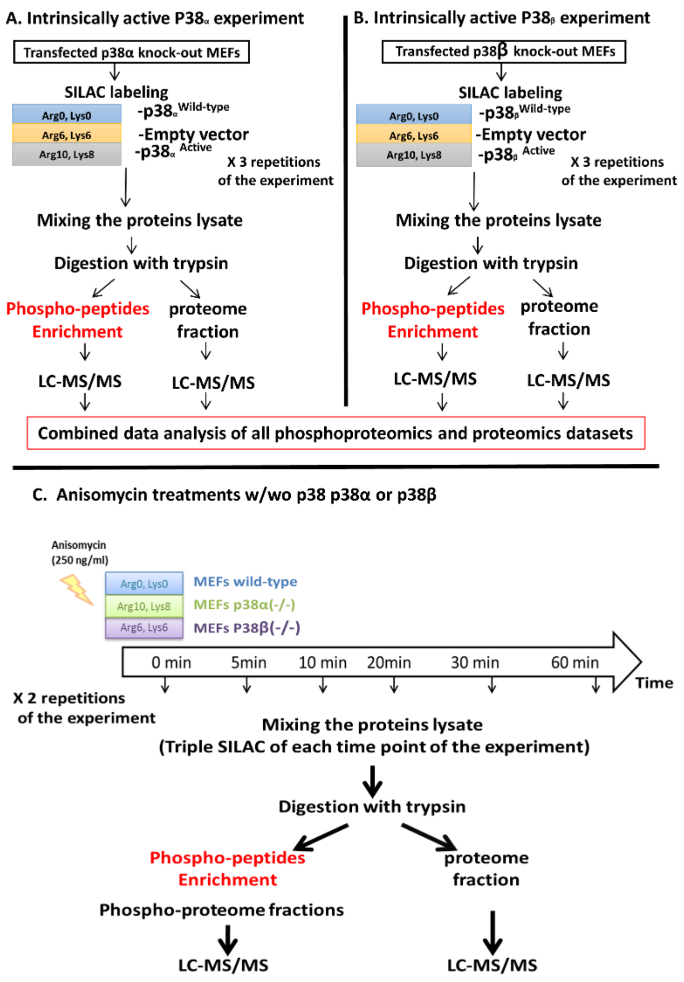

Supplementary Figure S1: **Experimental setups of the phosphoproteomics and proteomics analyses.**

(A-B) The p38 knockout MEFs, transfected with the p38 wildtype or intrinsically active p38 variants, were labeled using stable isotope labeled amino acids in cell culture (SILAC) [1,2]. Next, 1 mg of protein from each culture were mixed, digested with trypsin, and the phosphopeptides enriched. The phosphoproteomics analyses were performed in parallel with the regular proteomics analysis from the same extracts. (C) Similar to A-B above, SILAC labeling was used for following the differential influences of the two p38s on the effects of the anisomycin treatment. 1 mg of each protein extract were mixed, digested with trypsin, and analyzed in parallel for their phosphopeptides and proteins contents.

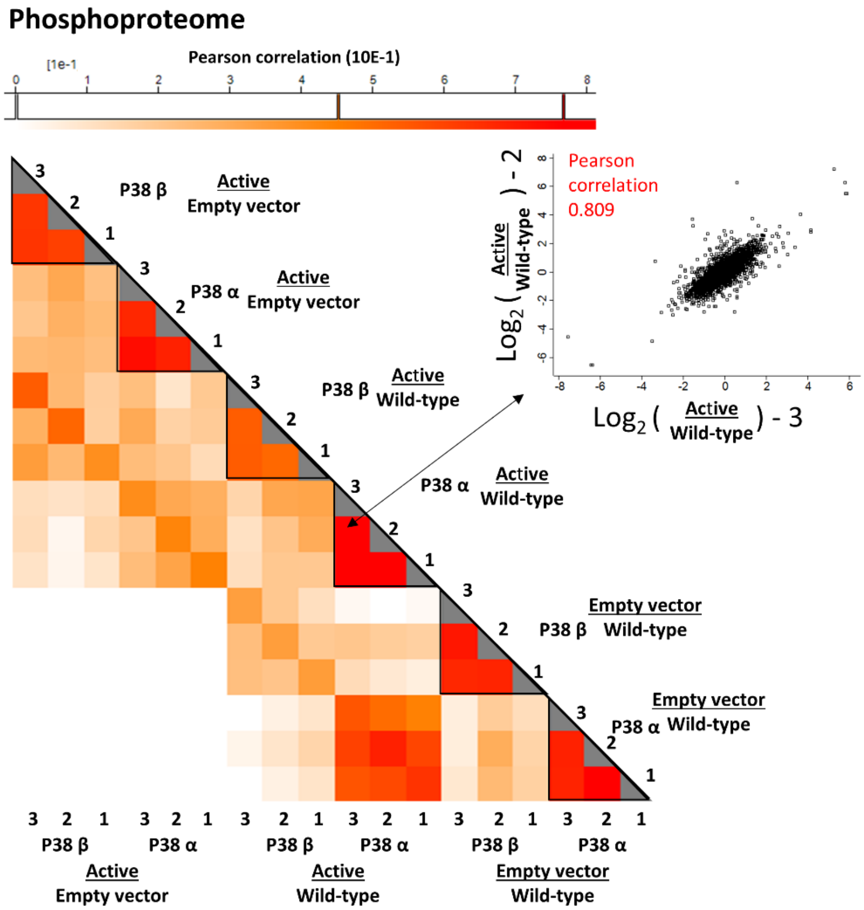

Supplementary Figure S2: **Highly reproducible phosphoproteome data was observed for triplicate analyses.** Heatmap of Pearson correlation coefficients were obtained for the different SILAC ratios of the phosphorylation sites detected in the different transfected MEFs. The ratios refer to the differences between the phosphorylation levels of the entire phosphor-peptidomes. Active/Wild-type ratios refer to the phosphorylation sites detected in the cells expressing the active variants, relative to the cells expressing the wildtype p38s. The averages of each ratio sets were used to generate the heatmap. The results of each of the three replicates (marked by the numbers: 1, 2, 3) of the two experiments (p38α and p38β) are displayed. The statistical analysis and the heatmap were produced by the Perseus software [3]. The MS data were interpreted by MaxQuant software version 1.5.0.25 [4].

A

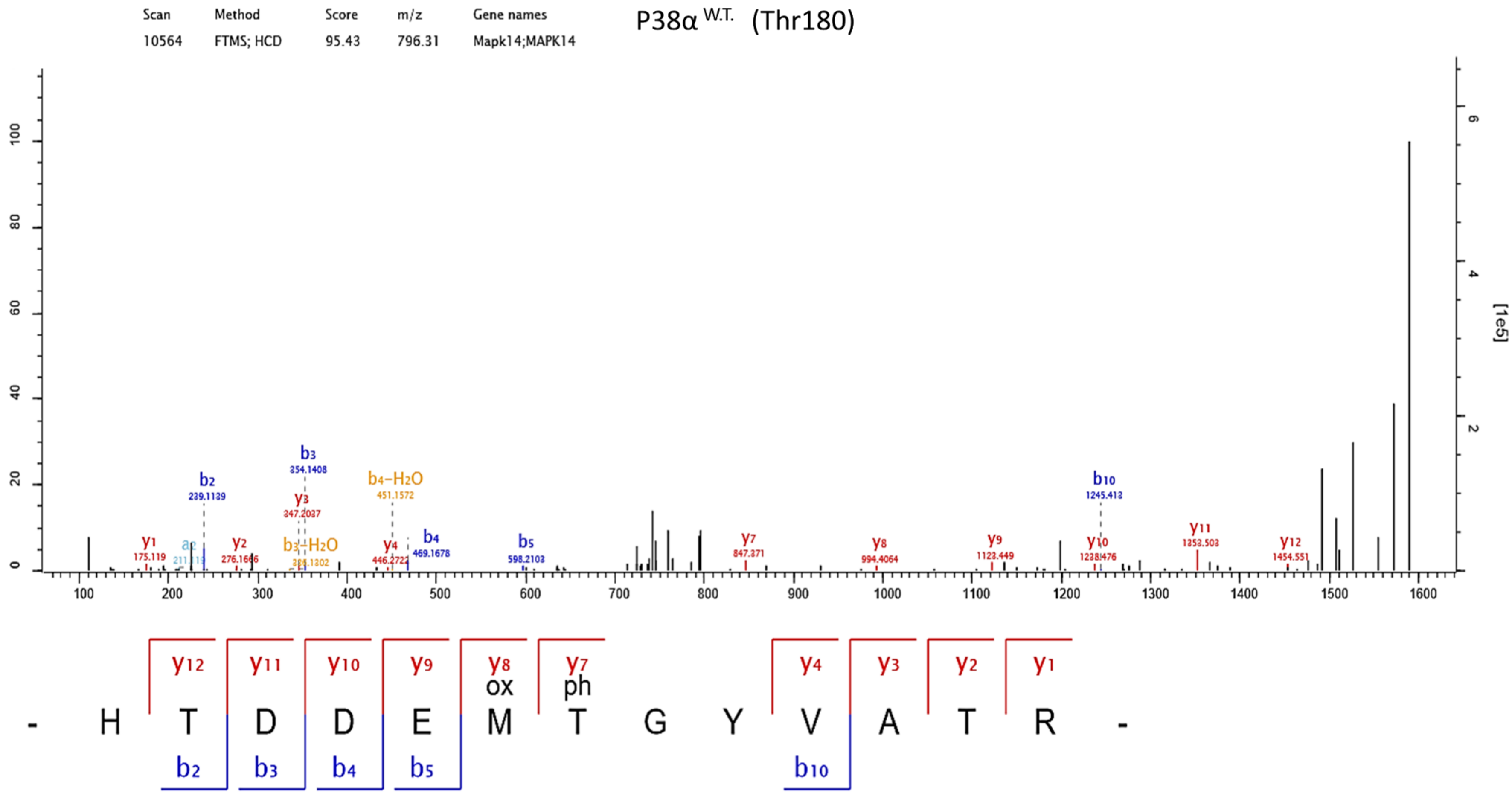
Scan 10564
Method FTMS; HCD
Score 95.43
m/z 796.31
Gene names Mapk14;MAPK14
P38α W.T. (Thr180)
[1e5]
y12 y11 y10 y9 y8 y7 y4 y3 y2 y1
ox
ph
- H T D D E M T G Y V A T R -
b2 b3 b4 b5 b10

B

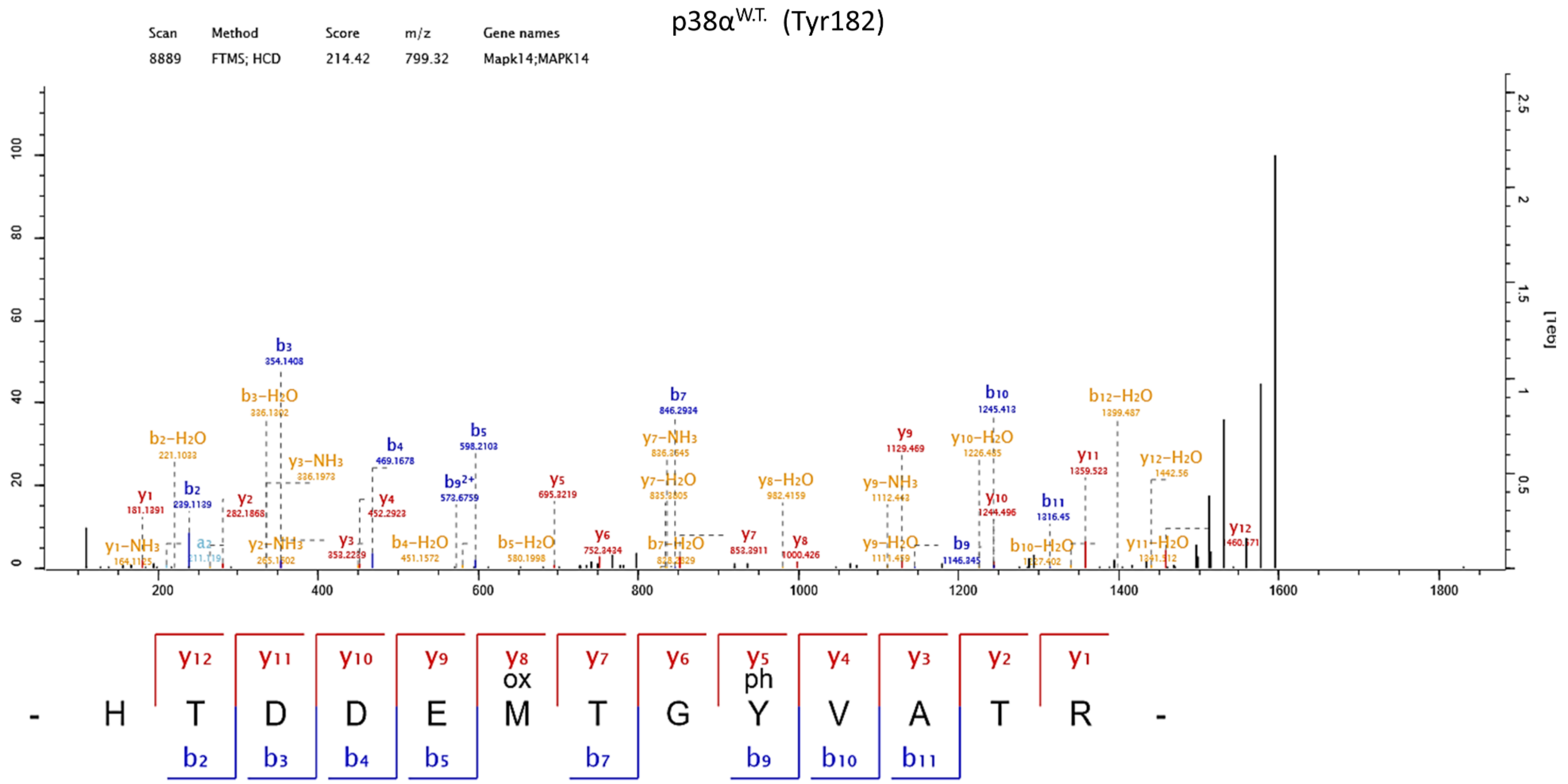
p38α W.T. (Tyr182)
Scan Method Score m/z Gene names
8889 FTMS; HCD 214.42 799.32 Mapk14;MAPK14
b3 354.1408
b3-H2O 336.1302
b2-H2O 221.1033
y3-NH3 336.1973
b4 469.1678
b5 598.2103
b7 846.2934
y7-NH3 836.2645
y7-H2O 835.2805
b10 1245.413
y10-H2O 1226.455
b12-H2O 1399.487
y9 1129.469
y11 1359.523
y12-H2O 1442.56
y1 181.1391
b2 239.1139
y2 282.1868
y4 452.2923
b9 2+ 573.6759
y5 695.3219
y8-H2O 982.4159
y9-NH3 1112.443
y10 1244.496
b11 1316.45
y1-NH3
a2
y2-NH3
y3 353.2259
b4-H2O 451.1572
b5-H2O 580.1998
y6 752.3434
b7-H2O
y7 853.2911
y8 1000.426
y9-H2O
b9 1146.345
b10-H2O
y11-H2O
y12 460.571
200 400 600 800 1000 1200 1400 1600 1800
0 20 40 60 80 100
0.5 1 1.5 2 2.5
[1e6]
y12 y11 y10 y9 y8 y7 y6 y5 y4 y3 y2 y1
ox ph
- H T D D E M T G Y V A T R -
b2 b3 b4 b5 b7 b9 b10 b11

C

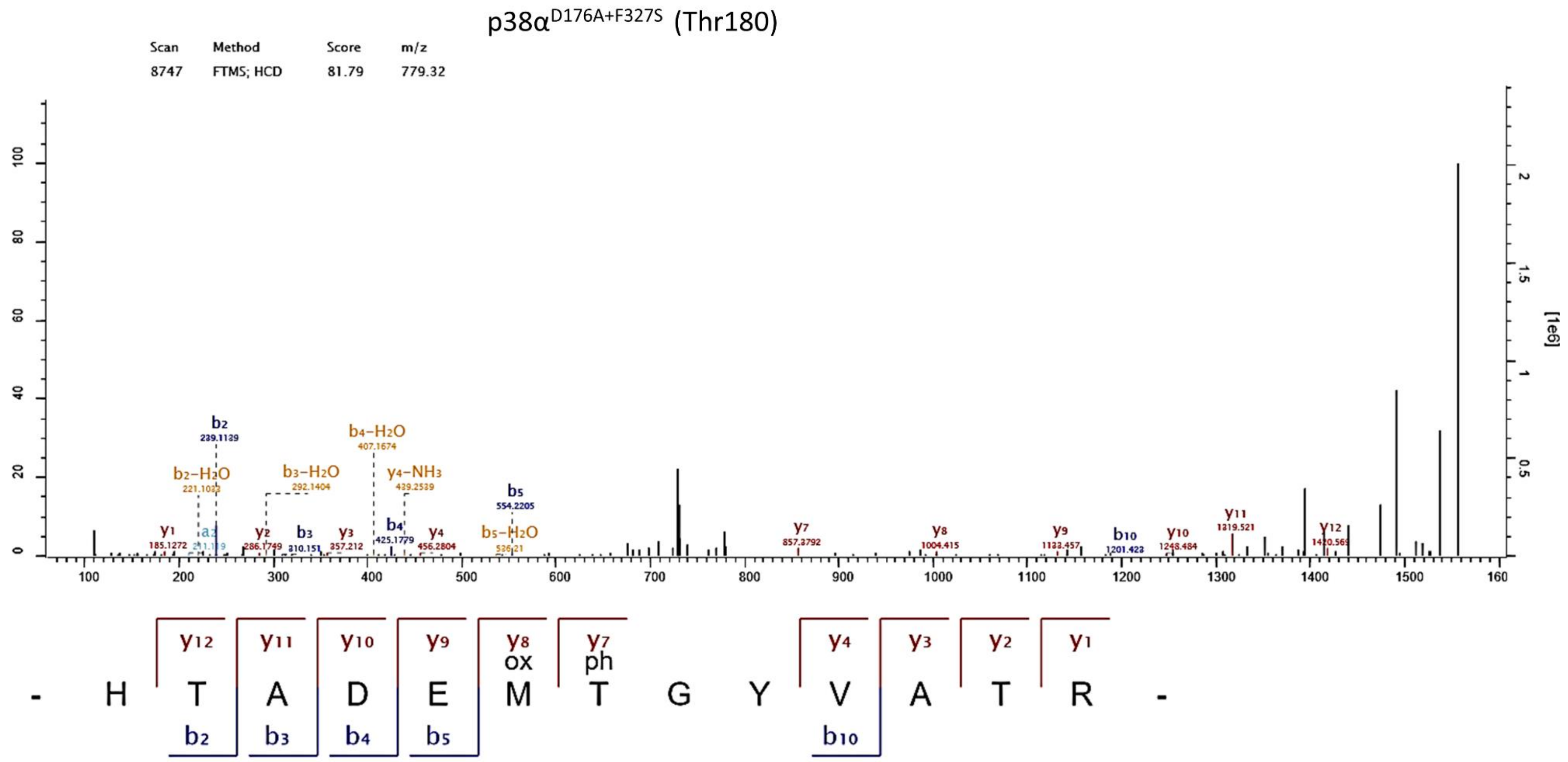
p38α D176A+F327S (Thr180)
Scan 8747
Method FTMS; HCD
Score 81.79
m/z 779.32
b2 239.1139
b2-H2O 221.1033
b3-H2O 292.1404
b4-H2O 407.1674
y4-NH3 439.2539
b5 554.2205
b5-H2O 536.21
y1 185.1272
y2 286.1749
b3 310.151
y3 357.212
b4 425.1779
y4 456.2804
y7 857.3792
y8 1004.415
y9 1133.457
b10 1201.423
y10 1248.484
y11 1319.521
y12
[1e6]
- H T A D E M T G Y V A T R -
y12 y11 y10 y9 y8 y7 y4 y3 y2 y1
ox ph
b2 b3 b4 b5 b10

D

p38β$^{W.T.}$ (Tyr182)

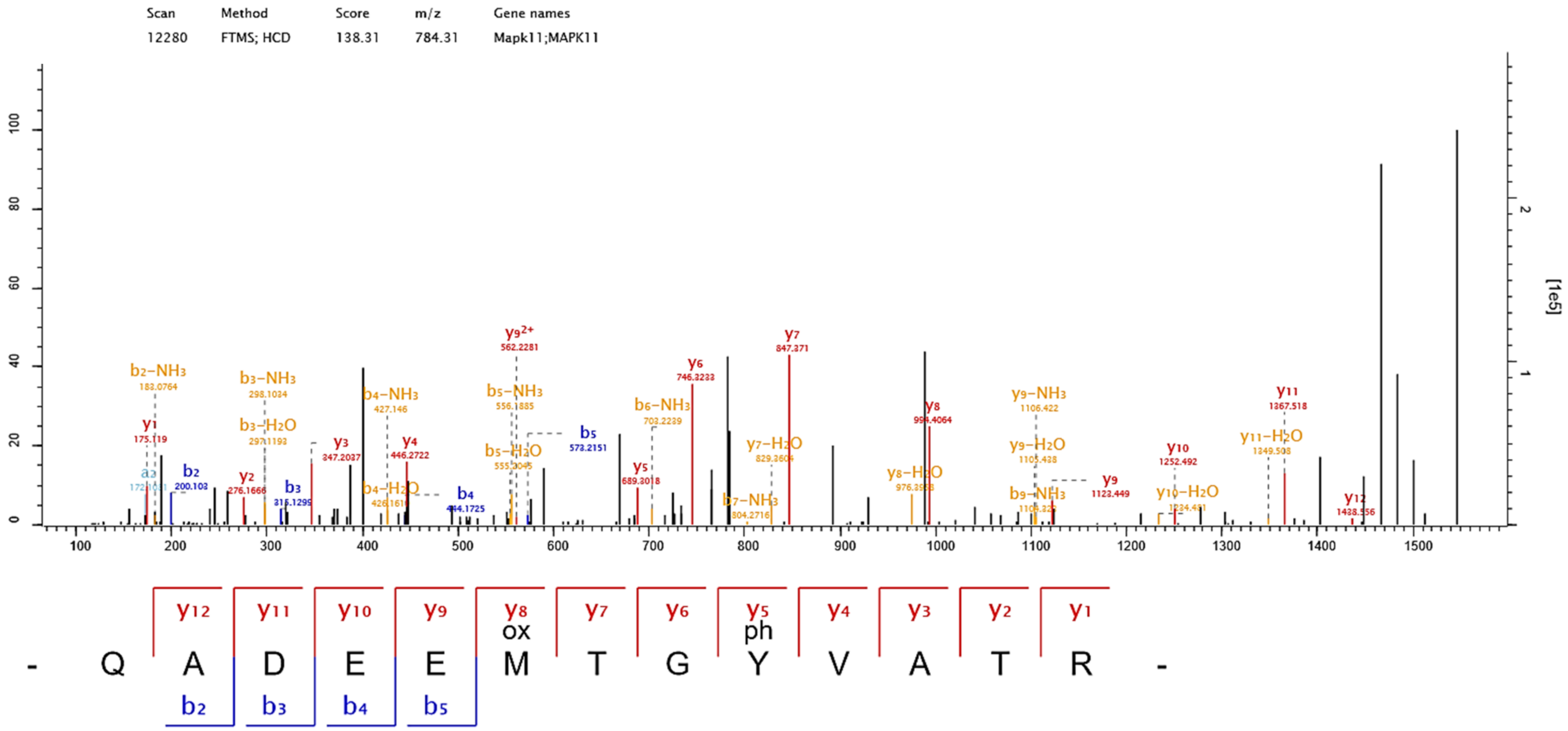

Supplementary Figure S3: **MS/MS spectra of the phosphorylation of p38α and p38β on their TGY motifs.**

Best MS/MS spectra of the phosphopeptides of p38 TGY motifs: (A) Phosphopeptide of the wildtype p38α, phosphorylated on Thr180; (B) phosphopeptide of the wild-type p38α, phosphorylated on Tyr182. (C) Phosphopeptide of p38α$^{D176A;F327S}$, phosphorylated on Thr180; (D) phosphopeptide of wild-type P38β, phosphorylated on Tyr182.

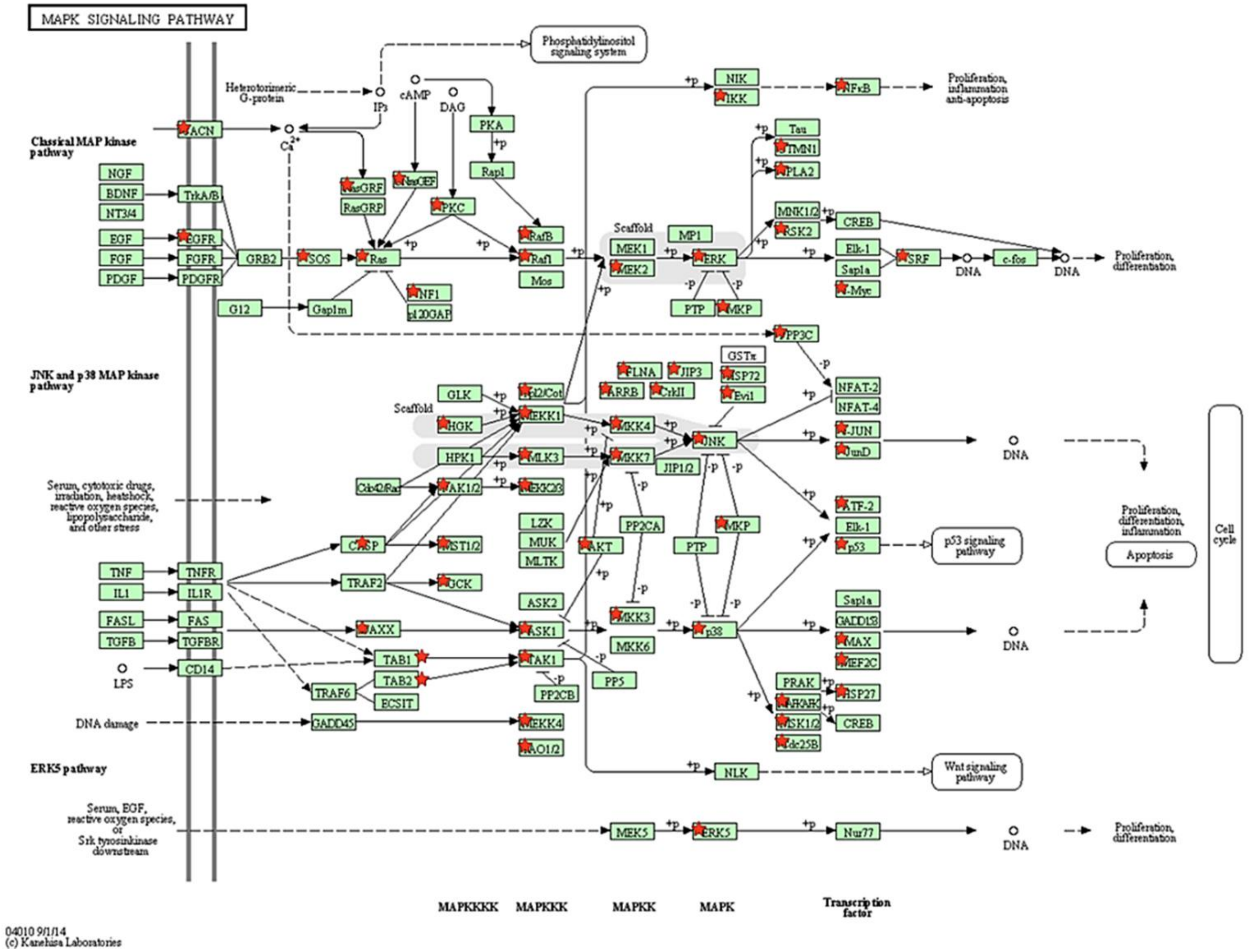

Supplementary Figure S4: **Identified phosphosites from the MAPK signaling pathway affected differentially by the anisomycin treatment.**

Red stars represent proteins identified with phosphorylation sites from the entire pathway of the MAPK signaling, as listed in the KEGG database [5]. The data is based on 404 phosphosites derived from 86 proteins identified here.

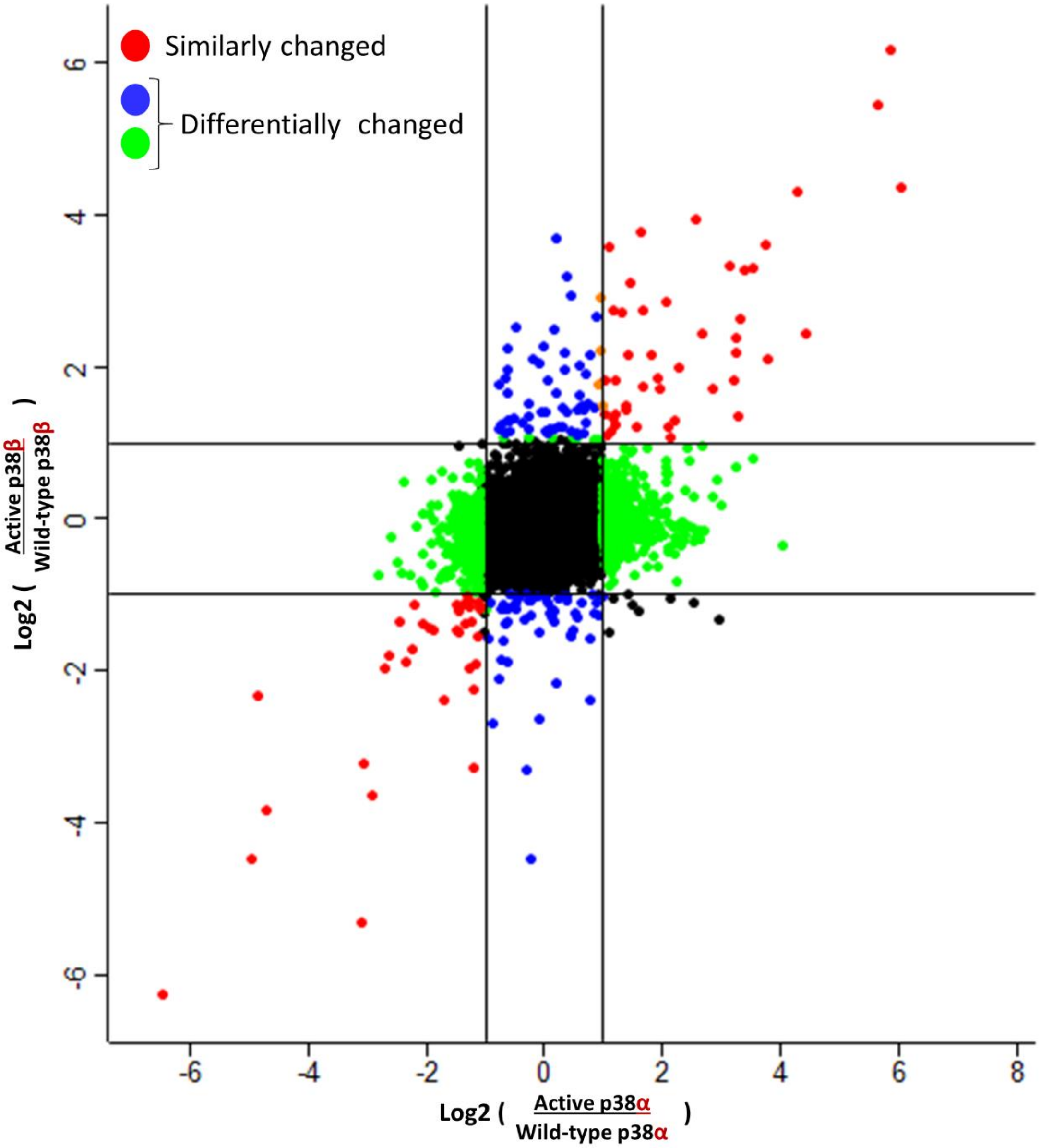

Supplementary Figure S5: **Differential effects of p38α and p38β on the phosphoproteome.**

Scatterplot of the log_2(active variant/wild-type ratio) of all the quantified phosphorylation sites of p38α versus p38β. Phosphorylation sites marked with red were similarly changed due to the expression of the intrinsically active p38α and p38β, and phosphorylation sites marked in blue or green were differentially changed due to expression of the intrinsically active p38α and p38β, respectively.

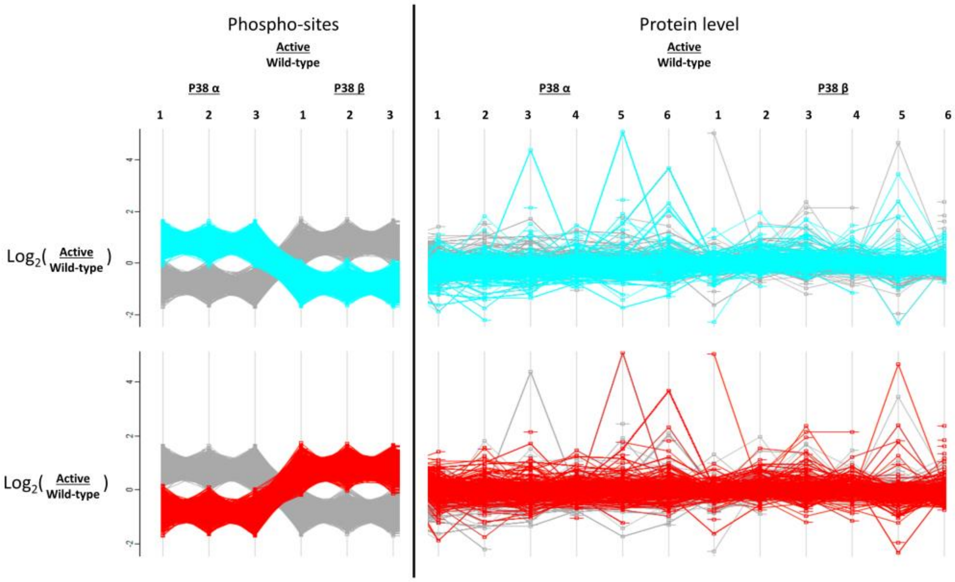

Supplementary Figure S6: **The source proteomes of most significantly changed phosphopeptides did not change in their levels due to the expression of the p38 active variants.**

Profile plot of log_2(active variant/wild-type ratio) of 2617 significantly changed phosphorylation sites and their source proteins. The blue lines in the profile plot indicate phosphopeptides that decreased in their ratio in p38β relative to p38α and the red lines indicate phosphopeptides that increased in their ratios between these p38s. The lines on the right indicate the ratios of the source proteins of these phosphopeptides, showing that these did not change significantly during the analysis. The gray lines are those labeled in blue or red in the opposite graphs.

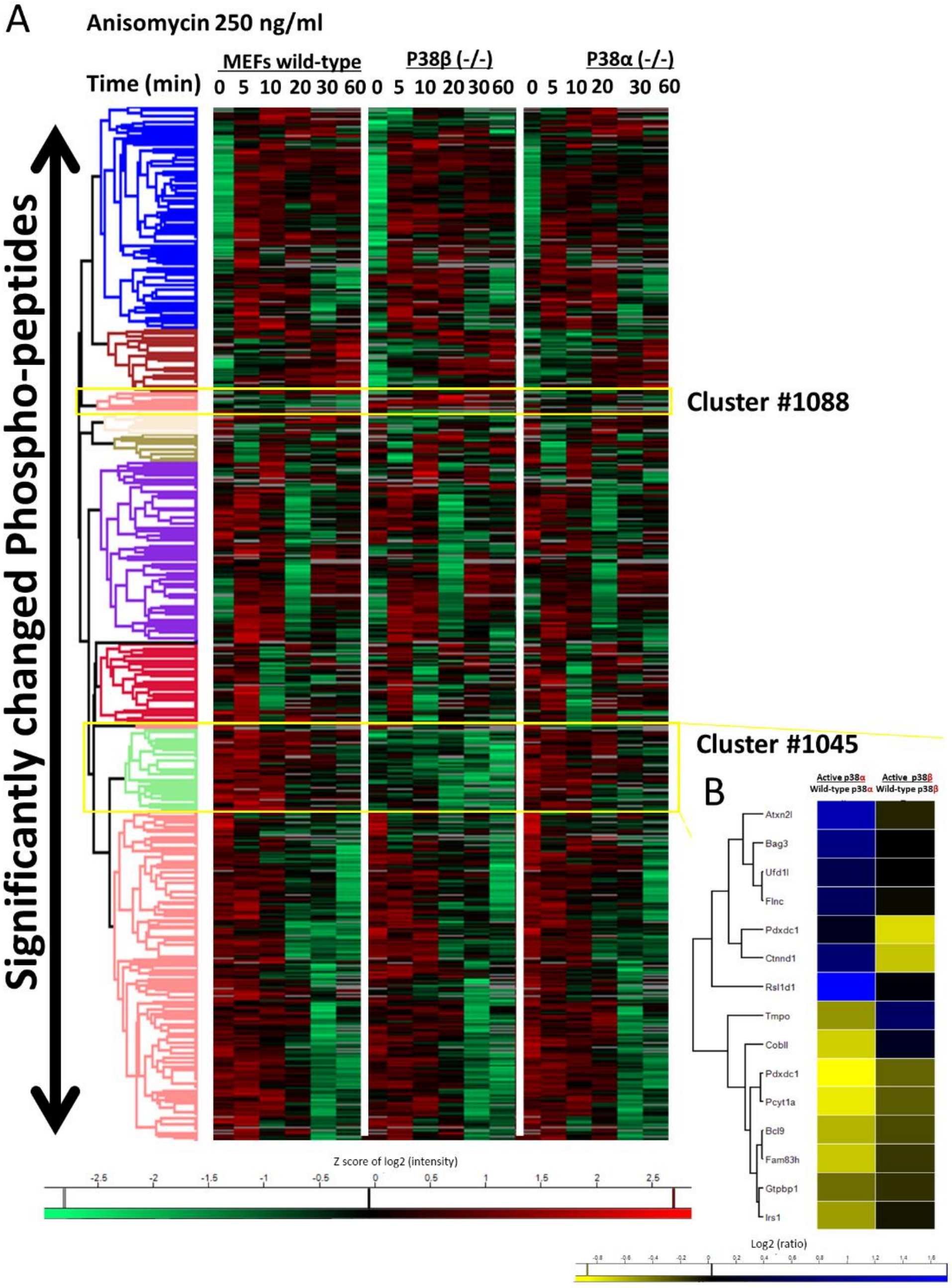

Supplementary Figure S7: **A fraction of the phosphorylation sites, significantly affected by anisomycin, follow different time course in the different cell lines.**

(A) A total of 1099 phosphorylation sites were defined as significantly changed by the ANOVA multi-sample test, in at least one cell line. These were divided to 9 clusters according to their dynamic profiles by hierarchical clustering. The number 1 and 2 on the right of the yellow blocks mark the clusters of phosphorylation sites that demonstrated differential patterns between the cell lines, irrespective of the treatment. (B) 14 phosphorylation sites from cluster #1045 were differentially regulated in the intrinsically active variant experiments. Heat map of log2 (ratio of active variant/wild-type). The statistical analysis was performed with the Perseus version 1.5.6.0 [3].

Phosphopeptides of HSP27 in MEFs treated with anisomycin

A

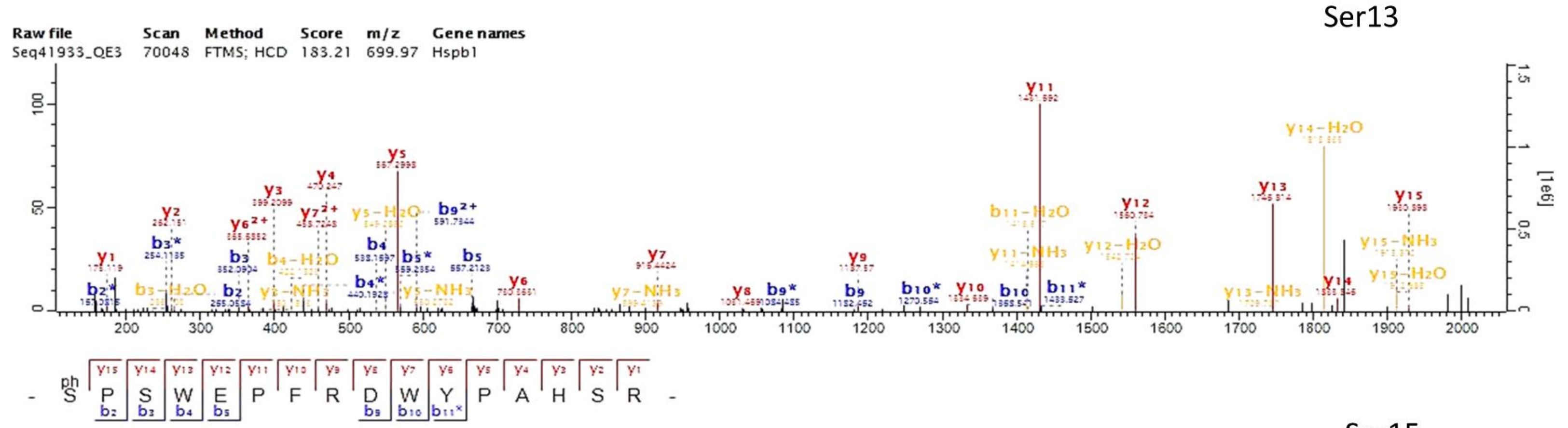

B

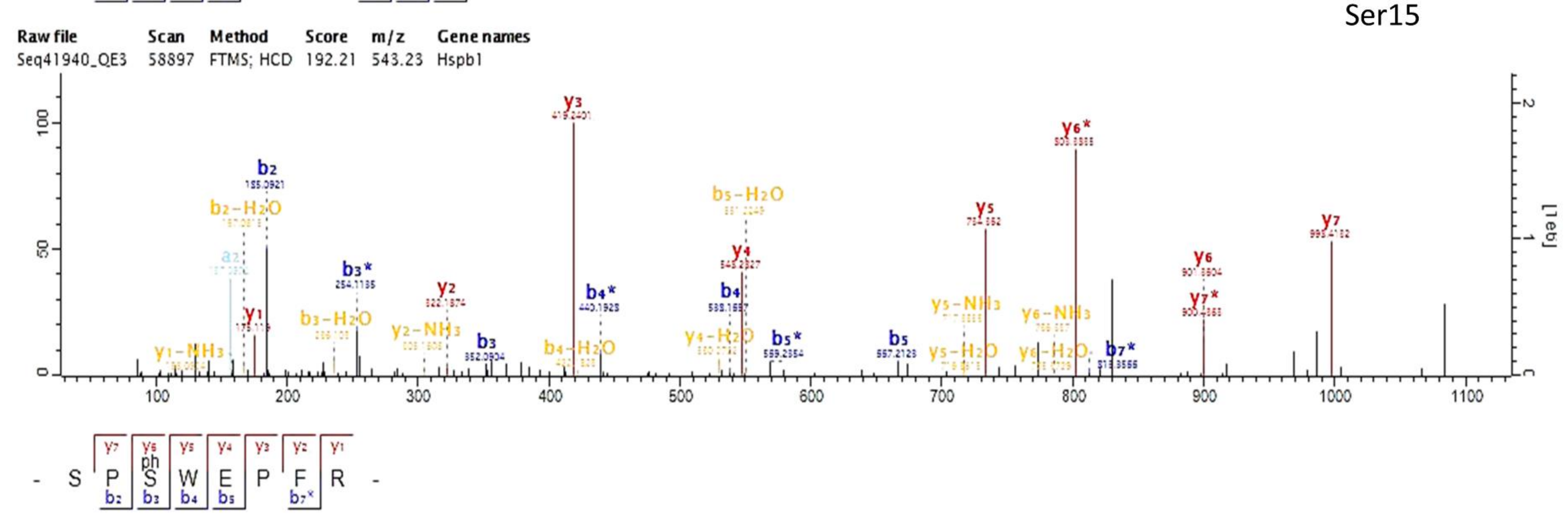

C

Ser86

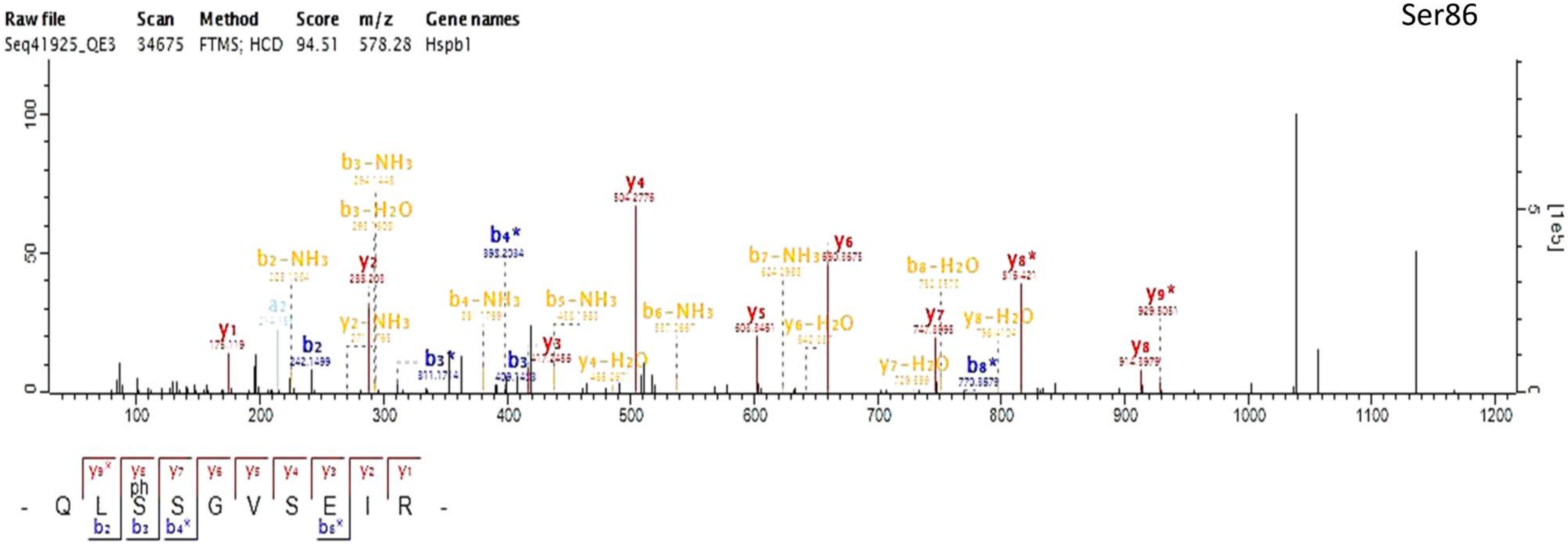

Raw file Scan Method Score m/z Gene names
Seq41925_QE3 34675 FTMS; HCD 94.51 578.28 Hspb1
100
50
0
[1e5]
b3-NH3
b3-H2O
y4
504.2776
b4*
398.2084
b2-NH3
y2
288.203
y6
660.3675
b7-NH3
b8-H2O
y8*
816.421
y9*
929.5051
y1
b2
242.1499
y2-NH3
b4-NH3
b5-NH3
b6-NH3
y5
y6-H2O
y7
y8-H2O
b3*
311.1714
b3
y3
y4-H2O
y7-H2O
b8*
770.3579
y8
100 200 300 400 500 600 700 800 900 1000 1100 1200
y9* y8 y7 y6 y5 y4 y3 y2 y1
ph
- Q L S S G V S E I R -
b2 b3 b4* b8*

Phosphopeptides of HSP27 in MEFs expressing the intrinsically active variants

D

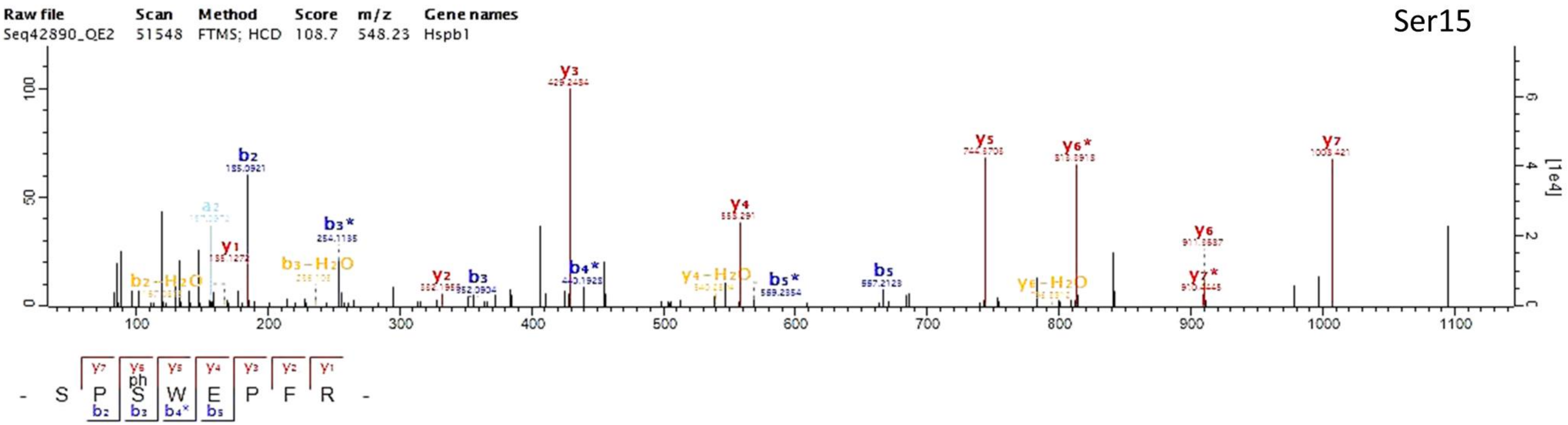

E

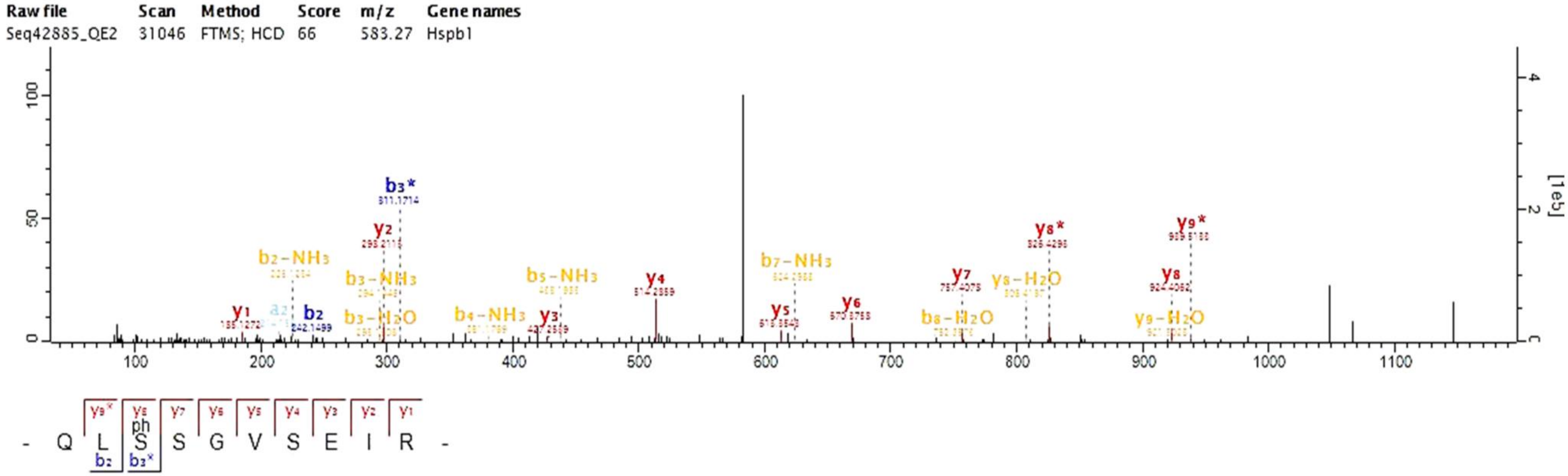

Supplementary Figure S8: **MS/MS spectra of the HSP27 phosphopeptides in MEFs treated with anisomycin or expressing the p38 active variants**.

The best MS/MS spectra of HSP27 phosphorylation sites: (A-C) Ser13 phosphorylation site in MEFs treated with anisomycin (A), Ser15(B), Ser86 (C). (D-E) phosphorylation sites in MEFs expressing the p38 intrinsically active variants, Ser15 (D), and Ser86 (E).

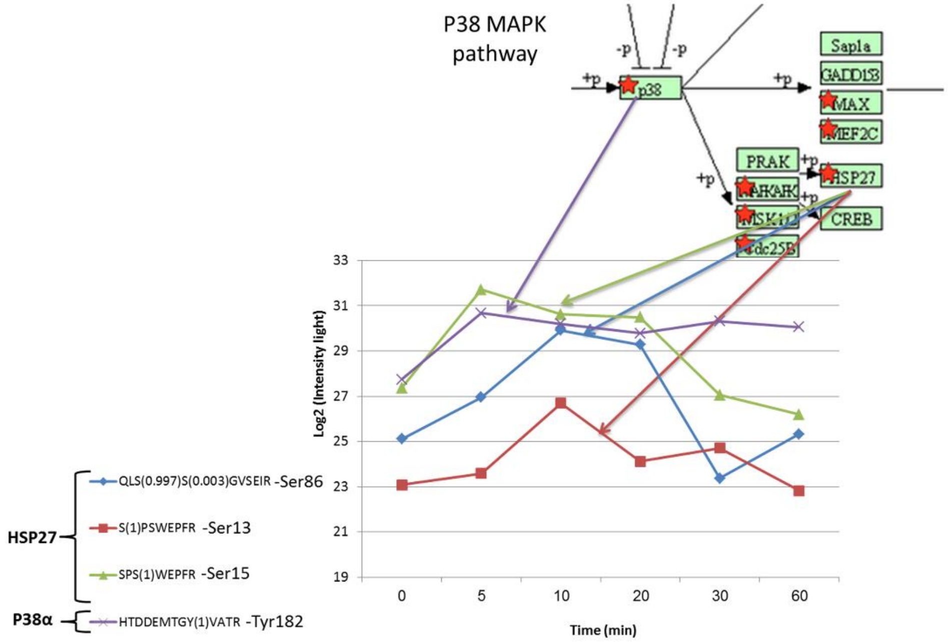

Supplementary Figure S9: **Time course of p38α and HSP27 phosphorylation sites induced by anisomycin of wildtype MEFs.**

Dynamics of the log2(intensity) of four phosphopeptides of p38α and HSP27 in the wild-type MEFs induced by anisomycin treatment. Red stars represent peptides identified in the anisomycin analysis with phosphorylation sites from the MAPK signaling pathway, as listed in the KEGG database [5]

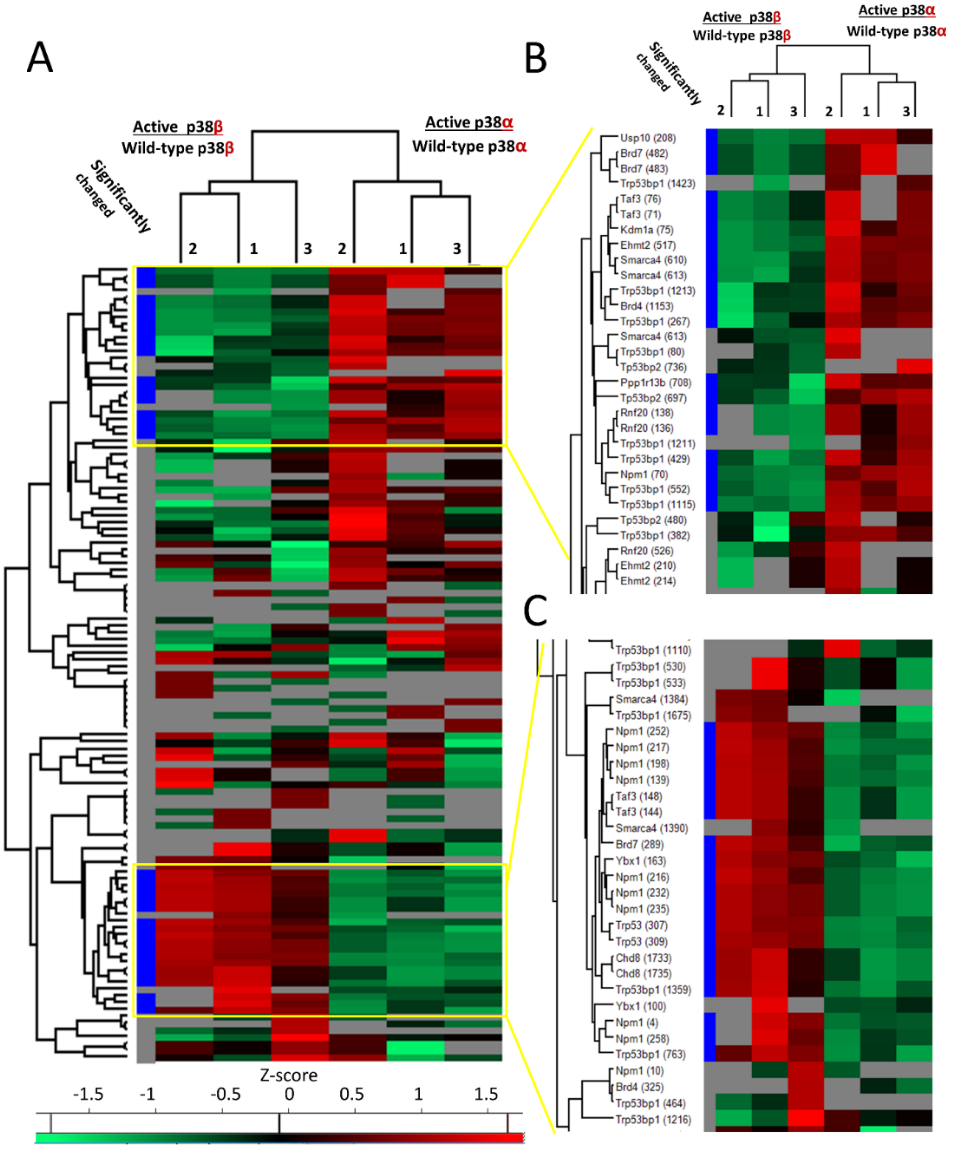

Supplementary Figure S10: **Phosphopeptides of p53-binding proteins significantly changed between the intrinsically active p38 datasets.**

(A) Heath map of log2(Active variant/Wildtype) after Z scoring of all phospho-peptides of p53-binding proteins. (B) Zoom into the heat map (log2(Active Variant/Wildtype) of phosphopeptides of p53-binding proteins, that were upregulated in p38α active variant. (C) Zoom into the heat map log2(Active Variant/Wildtype) of phosphopeptides of p53-binding proteins, that were upregulated in p38β active variant. (*Numbers in brackets next to the gene name are phosphorylation position within the protein).

Supplementary Tables S1-S3: “NaN" means peptides or proteins that were not quantified by the MaxQuant software.

References

1. Ong, S.-E.; Mann, M. A Practical Recipe for Stable Isotope Labeling by Amino Acids in Cell Culture (SILAC). *Nat. Protoc.* **2006**, *1*, 2650–2660, doi:10.1038/nprot.2006.427.

2. Ong, S.-E.; Blagoev, B.; Kratchmarova, I.; Kristensen, D.B.; Steen, H.; Pandey, A.; Mann, M. Stable Isotope Labeling by Amino Acids in Cell Culture, SILAC, as a Simple and Accurate Approach to Expression Proteomics. *Mol. Cell. Proteomics* **2002**, *1*, 376–386, doi:10.1074/mcp.m200025-mcp200.

3. Tyanova, S.; Temu, T.; Sinitcyn, P.; Carlson, A.; Hein, M.Y.; Geiger, T.; Mann, M.; Cox, J. The Perseus Computational Platform for Comprehensive Analysis of (Prote)Omics Data. *Nat Methods* **2016**, *13*, 731–740, doi:10.1038/nmeth.3901.

4. Cox, J.; Mann, M. MaxQuant Enables High Peptide Identification Rates, Individualized p.p.b.-Range Mass Accuracies and Proteome-Wide Protein Quantification. *Nat. Biotechnol.* **2008**, *26*, 1367–1372, doi:10.1038/nbt.1511.

5. Kanehisa, M.; Goto, S. KEGG: Kyoto Encyclopedia of Genes and Genomes. *Nucleic Acids Res.* **2000**, *28*, 27–30, doi:10.1093/NAR/28.1.27.
